# HPV-related oropharyngeal carcinoma remains infrequent over 25 years in a Brazilian Oral Pathology Center: A cross-sectional study with literature review

**DOI:** 10.4317/medoral.26462

**Published:** 2024-01-30

**Authors:** Adriana Aparecida Silva da Costa, Rafael Soares Guieiro, Ingrid Gomes de Oliveira, Thalita Soares Tavares, Daniela Pereira Meirelles, Evânio Vilela da Silva, Anderson Tangerino Ferreira da Silva, Jorge Esquiche León, Maria Cássia Ferreira de Aguiar, Patrícia Carlos Caldeira

**Affiliations:** 1 DDS, MSc, Department of Oral Pathology and Surgery, School of Dentistry, Federal University of Minas Gerais,, Belo Horizonte, MG, Brazil; 2 DDS, Department of Oral Pathology and Surgery, School of Dentistry, Federal University of Minas Gerais,, Belo Horizonte, MG, Brazil; 3 Undergraduate student. Department of Oral Pathology and Surgery, School of Dentistry, Federal University of Minas Gerais,, Belo Horizonte, MG, Brazil; 4 DDS, PhD. Oral Pathology, Department of Stomatology, Public Oral Health, and Forensic Dentistry, Ribeirão Preto Dental School (FORP/USP), University of São Paulo, Ribeirão Preto, SP, Brazil; 5 BIOMED. Oral Pathology, Department of Stomatology, Public Oral Health, and Forensic Dentistry, Ribeirão Preto Dental School (FORP/USP), University of São Paulo, Ribeirão Preto, SP, Brazil; 6 DDS, PhD. Department of Oral Pathology and Surgery, School of Dentistry, Federal University of Minas Gerais,, Belo Horizonte, MG, Brazil

## Abstract

**Background:**

The aim was to evaluate the frequency, clinicopathological features, and HPV status of oropharyngeal squamous cell carcinoma (OP-SCC) and benign HPV-related epithelial lesions of the oropharynx over the last 25 years. Moreover, a literature review was performed to investigate HPV frequency in OP-SCC samples diagnosed in Brazilian Centers.

**Material and Methods:**

A cross-sectional study analyzed OP-SCC, squamous papilloma, verruca vulgaris, and condyloma accuminatum, diagnosed from 1997 to 2021. HPV status of OP-SCC was determined by immunohistochemistry and “in situ” hybridization. Bivariate statistics were performed (p≤0.05). For the literature review, MEDLINE/PubMed, Web of Science, EMBASE, and Scopus were searched. Two independent reviewers assessed the studies for eligibility.

**Results:**

Cross-sectional: 211 OP-SCC (63.0%) and 124 benign lesions (37.0%) were included. OP-SCC frequency increased gradually over time, whereas benign lesions had steady trends. OP-SCC affected more males (*n*= 171; 81.0%), though the relative frequency in females rose over time. Smoking (*n*= 127; 60.2%) was common in OP-SCC. Nineteen OP-SCC (13.0%) were positive for HPV. HPV-positive and HPV-negative tumors had similar clinicopathological features (*p*>0.05). Benign lesions predominated in middle-aged (*n*= 32; 26.7%) women (*n*= 71; 57.3%), in the soft palate (*n*=101; 81.5%). Literature review: 32 studies were included, and in 60% of them, HPV frequency in OP-SCC was less than 25%.

**Conclusions:**

OP-SCC prevalence has been increasing, and it was mostly associated with smoking and alcohol rather than with HPV infection in Brazil. Benign lesions had a stationary frequency over the evaluated period.

** Key words:**Oropharynx, neoplasms, epidemiology, human papillomavirus viruses, squamous cell carcinoma.

## Introduction

The frequency of oropharyngeal squamous cell carcinoma (OP-SCC) has increased in the last decades, with 98,402 new cases and 48,143 new deaths worldwide in 2020 (1). Smoking, alcohol abuse, and human papillomavirus (HPV) are considered the main risk factors for OP-SCC (2).

The prevalence of HPV-related OP-SCC varies according to the geographic region and year of study (2). In the United States, 60% to 80% of OP-SCC are currently associated with HPV (3,4). In Europe, this percentage is also increasing, reaching 60% to 65% of prevalence in some countries such as Sweden and Denmark (5,6). Otherwise, a lower prevalence of HPV-related OP-SSC has usually been found in Brazil (7-9).

Over 200 subtypes of HPVs have been identified to date (10). In the oral and oropharyngeal mucosa, low-risk HPV infection (subtypes 2, 4, 6, 11, 13, 32, 40, 57) is usually associated with benign papillomatous lesions, whereas high-risk HPV infection (subtypes 16, 18, 33 and 35) is related to OP-SCC (11,12).

Interestingly, the oropharynx is not the preferred intra-oral site for the benign epithelial lesions associated with HPVs (11). Conversely to the increasing literature on OP-SCC, little is known about the current frequency of benign HPV-related lesions of the oropharynx (11,13,14).

Therefore, the present study aimed to explore a historical panorama of oropharyngeal HPV-related lesions at a reference center for Oral Pathology in Brazil. In addition, we reviewed the literature on HPV frequency in OP-SCC samples diagnosed in Brazilian Centers. That would be important facing the recent significant rise of OP-SCC cases in many countries, the great variability in the frequency of HPV-related OP-SCC around the world, and the poorly explored panorama of the benign HPV-related oropharyngeal lesions.

## Material and Methods

- Ethical approval

The study was approved by the Research Ethics Committee of Federal University of Minas Gerais, (10197919.9.0000.5149).

- Study design and sample selection

This cross-sectional study was performed and reported following the STROBE guidelines. Archives of the Oral Pathology Service of the School of Dentistry of Universidade Federal de Minas Gerais, Brazil, were reviewed from January 1997 to December 2021. The inclusion criteria were: histopathological diagnosis of a benign HPV-related epithelial lesion (squamous papilloma, condyloma accuminatum, or verruca vulgaris) or squamous cell carcinoma, located at the oropharynx (base of the tongue, soft palate, tonsils, and posterior pharyngeal wall). For p16 immunohistochemical analysis, paraffin blocks with scarce tissue were excluded.

Clinical and demographic data were collected from the biopsy charts and the total number of exams issued at the Oral Pathology Service each year was collected to calculate the relative frequency of the lesions.

- Histopathological evaluation of OP-SCC

OP-SCC routine slides were reviewed and classified as non-keratinizing (type I), keratinizing (type 2), or hybrid (type 3), as previously reported (15).

- p16 immunohistochemistry

The paraffin blocks of OP-SCC were retrieved and submitted to immunohistochemistry (IHC) for p16 (p16 INK4A, clone MX007, ready-to-use, Easypath Diagnósticos, Indaiatuba, SP, BR, code EP-12-52296).(4,16) The evaluation of p16 expression was conducted independently by two observers using an optical microscope. As previously established (4,16), tumors showing a strong and diffuse (> 70% of tumor cells) staining in both nuclei and cytoplasm were considered positive and were further submitted to “in situ” hybridization.

- Construction of tissue microarray

The tissue microarray was constructed with the p16-positive cases. Hematoxylin and eosin-stained sections were used to select representative areas of tumors. The TMA was constructed using a manually-built tissue matrix (Histopathology Ltd., Pécs, Hungary), with 2.0 mm diameter cylindrical cores. Tissue microarray slides were used for the “in situ” hybridization experiment.

- “In situ” hybridization (ISH) for HPV

“In situ” HPV DNA detection was performed with biotinylated probes Y1404 wide spectrum, including HPV genotypes 6, 11, 16, 18, 31, 33, 35, 39, 45, 51, and 52; and Y1443 GenPoint HPV, biotinylated DNA Probe targeting sequences of “high-risk” HPV genotypes 16, 18, 31, 33, 35, 39, 45, 51, 52, 56, 58, 59, and 68. Signal amplification was performed using K0620 (GenPoint, Dako, Carpinteria, CA, USA). The existence of dark/brown punctate (integrated) or diffuse (episomal) nuclear signals in the nuclei of tumor cells was interpreted as a positive reaction (8).

- Statistical analysis

Descriptive analysis was performed using the software SPSS version 26.0 (SPSS, Inc., Chicago, Illinois). Chi-squared test was used to compare the clinical and demographic features of patients with benign and malignant lesions as well as to compare the HPV-positive and HPV-negative OP-SCC cases. *p-value*s ≤ 0.05 were considered indicative of statistical significance.

- Literature review

A literature review was carried out to retrieve studies investigating HPV frequency in OP-SCC samples from Brazilian Centers. Inclusion criteria were: cross-sectional studies assessing OP-SCC diagnosed in Brazilian pathology services, whose HPV detection method has been clearly described and followed previously established criteria; English language; with no restriction on publication date. Case reports, reviews, and meeting abstracts were excluded as well as studies without confirmation of HPV, *in vitro* studies, studies with animals, studies in which Brazilian samples were analyzed together with samples from other countries, and studies in which oropharyngeal tumors were analyzed together with tumors from other anatomical sites.

The search was performed in MEDLINE/PubMed, Web of Science, EMBASE, and Scopus in July 2023, using the following search strategy: (“HPV” OR “papillomavirus” OR “papillomaviridae”) AND (“oropharynx” OR “oropharyngeal” OR “soft palate” OR “tonsils” OR “tongue base” OR “base of the tongue” OR “posterior pharyngeal wall”) AND (“cancer” OR “tumor” OR “neoplasms” OR “carcinoma” OR “squamous cell carcinoma”) AND (“Brazil” OR “Brazilian”). Two independent reviewers (A.A.S.C and D.P.M) assessed the titles, abstracts, and full texts for eligibility and data extraction.

Data extracted from the included articles were: authors, year of publication, institution where the study was done, period of investigation, sample size, methods for HPV detection, frequency of HPV-positive OP-SCC (positive % achieved in each method used and associated methods, when available).

## Results

- Cross-sectional

**Figure 1:**
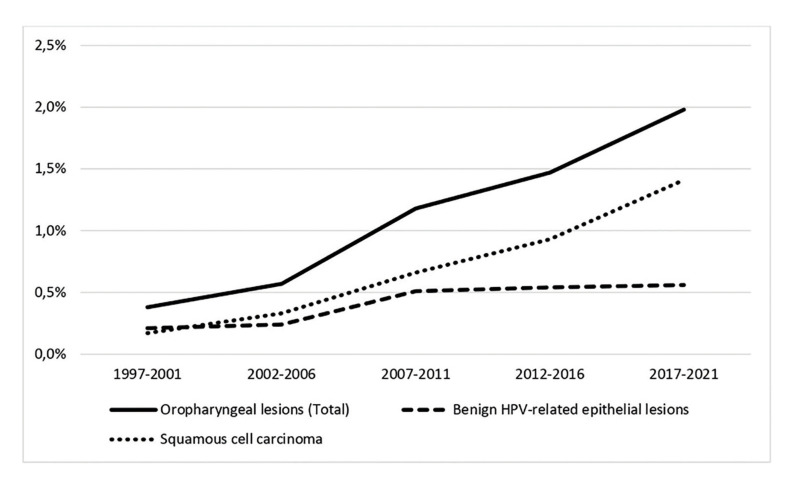
Relative frequency of histopathological diagnoses of oropharyngeal lesions, considering the total number of exams issued at the laboratory

335 (1.12%) oropharyngeal lesions were retrieved from 29,776 histopathological records, being 211 OP-SCC (63.0%), and 124 benign lesions (37.0%). Over 25 years, OP-SCC frequency increased gradually, whereas benign lesions had more steady trends. The relative frequency of oropharyngeal lesions over time is shown in Fig. 1. The clinical features of lesions are shown in Table 1, with the *p-value*s.

**Figure 2:**
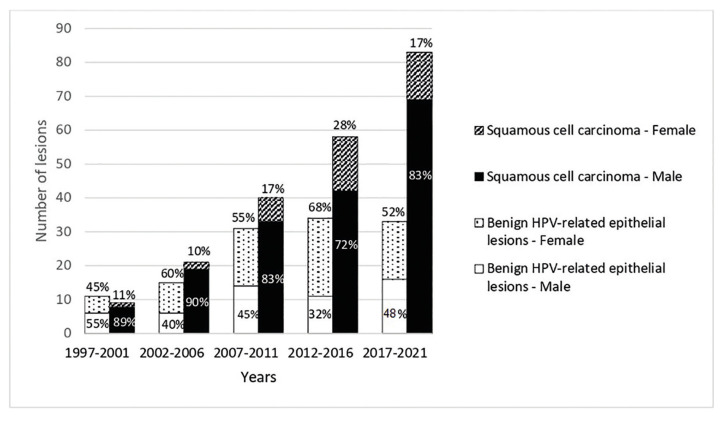
Relative and absolute frequency of the sex of the patients diagnosed with oropharyngeal lesions.

Patient sex data is demonstrated in Fig. 2. OP-SCC patients were most male (81.0%), in the sixth decade of life (40.0%). The mean age of patients with OP-SCC did not change significantly throughout the years (1997 to 2001: 60.2 years old; 2002 to 2006: 51.8; 2007 to 2011: 55.5; 2012 to 2016: 58.7; 2017 to 2021: 59.8). The youngest (21-30 years-old; *n*= 2) and oldest (81-90 years-old; *n*= 5) OP-SCC patients were diagnosed exclusively in the most recent years (2012 - 2021). The percentage of OP-SCC in females increased over the years, with a peak in 2012-2016. Female patients were mostly diagnosed at the 6th and 7th decade of life (62.5%) and had lower percentages of smoking and alcoholism than men (55.0% vs. 61.4% and 25.0% vs. 35.7%, respectively).

**Figure 3:**
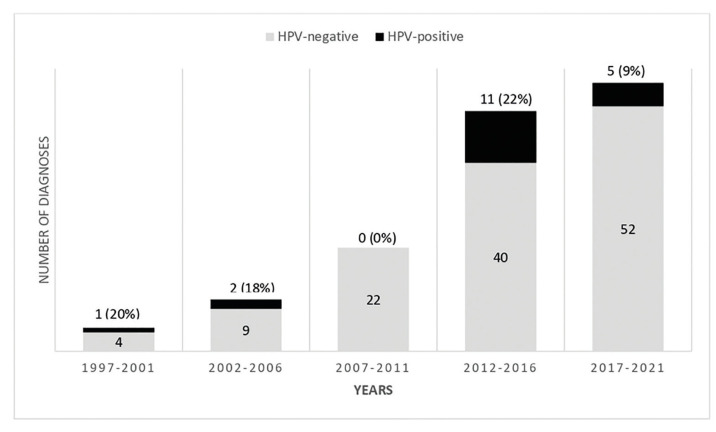
Frequency of HPV-negative and HPV-positive squamous cell carcinoma of the oropharynx.

One hundred sixty-one OP-SCC had available slides for histopathological evaluation, from which 80 (49.7%) were keratinizing, 71 (44.0%) were nonkeratinizing, and 10 (6.3%) were hybrid. One hundred and forty-six cases (69.2%) had available paraffin blocks for p16 testing, from which 19 (13.0%) were positive. DNA ISH revealed the presence of high-risk HPV DNA in all p16 positive cases. The frequencies of HPV-positive and negative OP-SCC over time are shown in Fig. 3. The absolute frequency of HPV-positive OP-SCC varied over the 25 years, with a peak in 2012-2016 (*n*=11). The percentage of HPV-positive OP-SCC among all OP-SCCs ranged from 18 to 22% over the years, except in 2007-2011 (no HPV-positive OP-SCC), with a decrease in 2017-2021 (9%).

Demographic and clinicopathological features of the HPV-positive OP-SCC are presented in Table 2. Patients were predominantly males (79.0%), with an age range from 32-85 years-old and mean age of 58 years. The age extremes - the youngest and oldest patients - were diagnosed in 2005 and 2013, respectively. From the cases with available information (*n*= 15), 12 patients (80.0%) were current or former smokers, and three (20.0%) had never smoked. Regarding alcohol use, from the cases with available information (*n*= 13), nine patients (70.0%) were current or former drinkers, and four (30.0%) have never drunk. No statistical difference (*p*>0.05) was noted between HPV-positive and HPV-negative patients regarding the clinicopathological variables.

Among benign lesions, squamous papilloma (*n*= 113; 91.1%) was the most common diagnosis, followed by verruca vulgaris (*n*= 10; 8.1%) and condyloma accuminatum (*n*= 1; 0.8%). These lesions were more frequent in females (57.3%), in the fifth decade of life (26.7%). The increase in the frequency of benign lesions over the years occurred in both males and females. The mean age of patients with benign lesions raised over time (1997 to 2001: 39.0 years old; 2002 to 2006: 38.4; 2007 to 2011: 48.0; 2012 to 2016: 50.7; 2017 to 2021: 49.0), with a higher prevalence of patients older than 41 years old in the last 15 years (2007-2021; *n*= 69, 57.5%).

- Literature review

**Figure 4:**
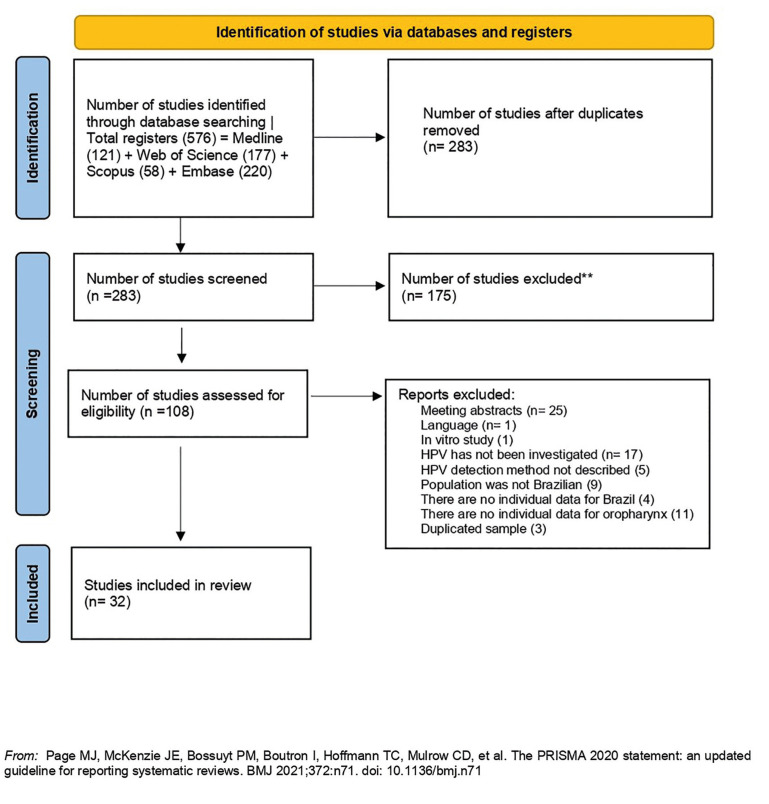
RISMA flow diagram for studies selection.

Fig. 4 shows the PRISMA flow diagram for study selection. 576 articles were retrieved. The eligibility criteria were applied, and 32 articles were included (Table 3, Supplement 1). The frequency of HPV in OP-SCC ranged from 0 to59.1%. A frequency less than 25% was reported in 60.0% (*n*=19) of the studies, ten of which (31%) showed a frequency ≤ 10% when different detection methods were associated. The pathology centers were located in the states of São Paulo (*n*=22), Rio de Janeiro (*n*=2), Mato Grosso (*n*=1), Minas Gerais (*n*=1), Ceará (*n*=1), Rio Grande do Sul (*n*=1), Goiás (*n*=1), Distrito Federal (*n*=1), and two studies encompassed samples from more than one state. Considering studies with larger sample sizes, the highest frequency of HPV in OP-SCC was observed in A.C. Camargo Cancer Center, São Paulo (59.1%), and the lowest frequency was seen in Brazilian National Cancer Institute, Rio de Janeiro (6.1%). The association of two or more methods for HPV detection (IHC plus PCR or IHC plus ISH) was commonly used (*n*=13; 41.0%). PCR as the only detection method was used in 10 studies (31.2%), whereas nine (28.1%) employed IHC exclusively.

## Discussion

The global incidence of OP-SCC has increased in recent years, and this steep rise has been mainly related to HPV infection in some regions, such as the United States, northern Europe, and Australia (1,5,6,12). The present study revealed an increase in the absolute and relative frequency of OP-SCC diagnosed at a Brazilian public oral and maxillofacial pathology center over the past 25 years. However, facing the very low frequency of HPV-positive tumors found herein, this increase could not be attribuTable to HPV-associated OP-SCC. In accordance, the literature review showed that most Brazilian studies presented a frequency ≤25% of HPV-positive OP-SCC, irrespective of the different methods used for HPV detection and sources of the samples (medical pathology center, tertiary health care). Similar to our results, a recent study demonstrated a prevalence of HPV-positive OP-SCC around 18% in South American patients (17).

The geographical variation in the prevalence of HPV-positive OP-SCC may be explained by the predominance of distinct risk factors in each region. In North America and Europe, changes in sexual behavior, including large numbers of lifetime oral sexual partners, seem to be responsible for a higher frequency of HPV-associated OP-SCC. Otherwise, in Brazil, these changes in sexual practices are not yet expressive (7,18). Individual sexual behaviors of Brazilians may differ from other populations with a high percentage of HPV OP-SCC.

Previous studies and the present one concur that OP-SCC remains more prevalent in men than in women (19,20). However, it is noteworthy that the rise of females affected by OP-SCC over time is demonstrated herein. Of relevance, women are diagnosed at an older age, with a lower frequency of smokers and drinkers than men. Some previous studies have reported a higher prevalence of OP-SCC in women (21,22), despite considering absolute frequency data in their assessments. Regarding the age of OP-SCC patients, although no important change in the average age of OP-SCC was noted over the years, the age extremes - including two very young patients - were diagnosed in recent years, suggesting a new nuance of the patient’s profile. Future research should investigate other environmental and genetic risk factors associated with HPV-unrelated OP-SCC in non-smoker and non-drinker women as well as in very young patients.

It is now well established that HPV-associated OP-SCC is a distinct condition from HPV-unassociated OP-SCCs (23), commonly affecting younger patients never exposed to alcohol and tobacco use (7,10). In the current study, even considering the sample size limitation, the demographic features of the HPV-positive OP-SCC patients were similar to those with HPV-negative tumors, corroborating other Brazilian studies (2,9,24). Importantly, a history of smoking and alcohol consumption was frequent in a great proportion of HPV-positive OP-SCC patients, thus the concomitant role of tobacco and alcohol with HPV infection in the etiopathogenesis of such tumors cannot be ruled out.

Contrary to OP-SCC, the relative frequency of benign epithelial lesions of the oropharynx was kept sTable over the years. No previous study reported a similar analysis, impairing comparisons or further conclusions. Such lesions have been mostly described in women (13), as found herein; however, males represented a great proportion as well, with no important change in this scenario over time. The present results demonstrated that older patients (more than 41 years) are getting more commonly diagnosed with benign lesions of the oropharynx in recent years, corroborating the results of Pina *et al* (11).

In the current study, benign lesions were most common in the soft palate, in line with previous reports (11,25). For OP-SCC, tonsils have been cited as the preferred site (5,6,24,26), however a non-specified sub-site predominated in the current study. The difficulty in determining the lesion epicenter due to the advanced cancer stage (26) probably interfered with this result. Importantly, the clear distinction of the other clinical features, such as clinical appearance, time of onset, and presence of symptoms, indicate that from the clinical standpoint, a benign oropharyngeal lesion should not be mistaken for an OP-SCC. Of notice, an early-stage OP-SCC shall appear clinically as a leukoplastik, erytroplastik, or leukoerytroplastik lesion that should not be misdiagnosed (27). In this regard, it is important to emphasize that there are no reports of HPV-positive premalignant lesions preceding OP-SCC, in contrast to HPV-induced cervical and anogenital neoplasms, which are usually preceded by precancerous lesions (28). In 1996, Fornatora *et al*. described a subtype of oral epithelial dysplasia (named koilocytic dysplasia) that harbors HPV DNA (29). Nevertheless, a small number of cases have been reported so far, and there is a gap in knowledge regarding this condition, especially its potential for malignant transformation (30). Thus, the benign epithelial lesions evaluated herein are not expected to precede OP-SCC.

The limitations of the present study are: results reflect the characteristics of lesions submitted to the histopathological exam only; lesions located in oropharyngeal sub-sites less accessible for the dentist should not be properly represented; not all cases of OP-SCC were available for HPV testing.

In conclusion, OP-SCC prevalence increased over the last 25 years, and it was mostly associated with smoking habit and alcohol intake rather than with HPV infection. An upward trend of occurrence of OP-SCC in females throughout the years was evidenced. Benign epithelial lesions had a stationary frequency over 25 years. Literature review reveals a low frequency of HPV in OP-SCC in most Brazilian studies.

## Figures and Tables

**Table 1:** Clinical features of squamous cell carcinoma and benign HPV-related epithelial lesions of the oropharynx, Brazil (1997-2021).

Variables		OP-SCC (n=211) No. of lesions (%)	Benign HPV-related epithelial lesions (n=124) No. of lesions (%)
Clinical appearance*	Ulcer	113 (53.7%)	0 (0.0%)
	Tumor	26 (12.3%)	2 (1.6%)
	Plaque	6 (2.8%)	1 (0.8%)
	Nodule	2 (0.9%)	23 (18.5%)
	Papule	0 (0.0%)	30 (24.2%)
	Other	4 (1.9%)	31 (25.0%)
	Missing	60 (28.4%)	37 (29.8%)
Time of onset*	0-6 months	106 (50.2%)	36 (29.0%)
	> 6 months	18 (8.5%)	20 (16.1%)
	Uncertain	74 (35.1%)	60 (48.4%)
	Missing	13 (6.2%)	8 (6.5%)
Symptoms*	Yes	140 (66.3%)	3 (2.4%)
	No	59 (28.0%)	111 (89.5%)
	Missing	12 (5.7%)	10 (8.1%)
Subsite*	Oropharynx (not specified)	96 (45.6%)	11 (8.8%)
	Soft palate	75 (35.5%)	101 (81.5%)
	Base of the tongue	26 (12.3%)	0 (0.0%)
	Tonsils	14 (6.6%)	12 (9.7%)
Smoking status	Current	127 (60.3%)	NA
	Previous	23 (9.1%)	NA
	No	21 (8.5%)	NA
	Missing	40 (22.1%)	NA
Alcohol use	Current	71 (33.6%)	NA
	Previous	39 (18.5%)	NA
	No	45 (21.3%)	NA
	Missing	56 (26.5%)	NA

NA: not available. *statistically significant (p<0.01), chi-squared test.

**Table 2:** Clinicopathological and demographic features of patients with HPV-positive* oropharyngeal squamous cell carcinoma diagnosed at a Brazilian oral pathology center from 1997 to 2021.

Case	Year	Sex	Age	Clinical aspect	Size (mm)	Subsite	Smoking status	Alcohol use	Histopathological classification
1	2000	M	46	Ulcer	20	NS	NA	NA	Nonkeratinizing
2	2005	M	32	Ulcer	30	NS	NA	NA	Nonkeratinizing
3	2006	M	60	Ulcer / tumor	20	Soft palate	NA	NA	Nonkeratinizing
4	2012	M	39	NA	140	NS	No	No	Nonkeratinizing
5	2013	F	85	NA	70	NS	Yes	NA	Nonkeratinizing
6	2013	M	61	NA	30	NS	Former	Former	Nonkeratinizing
7	2014	M	52	Other	20	Tongue base	Yes	Yes	Nonkeratinizing
8	2014	F	64	Ulcer	7	Soft palate	Yes	No	Nonkeratinizing
9	2015	M	61	NA	NA	NS	Yes	Former	Nonkeratinizing
10	2015	M	70	Ulcer	30	Soft palate	Yes	Yes	Keratinizing
11	2015	F	46	Tumor	50	NS	No	No	Nonkeratinizing
12	2016	M	68	NA	60	Soft palate	NA	NA	Hybrid
13	2016	M	63	NA	>50	NS	Yes	Yes	Nonkeratinizing
14	2016	M	57	NA	10	Soft palate	No	No	Nonkeratinizing
15	2018	M	53	Ulcer	15	Tongue base	Former	Former	Nonkeratinizing
16	2018	M	64	Ulcer	25	Soft palate	Former	Yes	Nonkeratinizing
17	2019	M	55	NA	70	NS	Yes	Yes	Nonkeratinizing
18	2021	M	61	NA	50	Soft palate	Former	Yes	Nonkeratinizing
19	2021	F	62	Ulcer	10	Soft palate	Yes	NA	Nonkeratinizing

* determined by immunohistochemistry and "in situ" hybridization. Abbreviations: M: Male. F: Female. NA: Not available. NS: Not specified.

**Table 3:** Human papillomavirus frequency in samples of oropharyngeal squamous cell carcinomas (OP-SCC) from Brazil.

Author	Year	Institution	Study period	Sample size	HPV detection	OP-SCC HPV+
Miguel et al.	1998	A.C. Camargo Cancer Center and HNS Department of School of Medicine (USP), SP	1995-1996	11	PCR	36%
Cortezzi et al.	2004	Arnaldo Vieira de Carvalho Institute and School of Medicine (FAMERP), SP	NA	21	PCR	14%
De Freitas Cordeiro-Silva et al.	2012	Santa Rita de Cássia Hospital, ES, and Araújo Jorge Hospital, GO	NA	4	PCR	0%
Cantarutti et al.	2014	Anatomopathology Division of the University Hospital of Brasilia, DF	2005-2011	13	PCR	0%
Lopez et al.	2014	Multicenter study. Patients recruited from three Brazilian cities (Goiânia, Rio de Janeiro, and São Paulo)	1998-2009	91	PCR	7%
Soares et al.	2014	Oral Oncology Center, UNESP, SP	2005-2007	37	ISH, PCR	32%
Hauck et al.	2015	Brazilian National Cancer Institute, RJ	2007-2009 Mar - Nov 2011	71	IHC, ISH, PCR	IHC: 17% | PCR: 15% | ISH: 11% IHC+ISH+PCR= 10%
Marques et al.	2015	Department of Otorhinolaryngology at Santa Casa de São Paulo, SP	Apr - Dec 2012	14	PCR	7%
Queiroz et al.	2014	Mato Grosso Cancer Hospital - Cuiabá, MT	NA	22	ISH, IF	ISH: 59% | IF: 54%
Piña et al.	2016	Pathology Department at UNICAMP, SP	NA	13	IHC, ISH	IHC - 31% | ISH - 0%
Betiol et al.	2016	Pathology Centers of Santa Casa de São Paulo and Cancer Institute of São Paulo (ICESP), SP	1991-2012	28	IHC, PCR	IHC: 14% | PCR: 14% IHC+PCR= 11%
Costa et al.	2016	Oncology Service of the University of Campinas Teaching Hospital, SP	2005-2013	94	IHC, ISH	IHC: 5% | ISH: 1% IHC+ISH=1%
Petito et al.	2017	Cancer Reference Center in Goiás (Associacão de Combate ao Câncer em Goiás), GO	2005-2007	43	PCR	26%
Anantharaman et al.	2017	Brazilian Head and Neck Genome Project, SP	2002-2015	171	IHC, PCR	IHC: 10% | PCR: 10% IHC+PCR= 4%
Barros-Filho et al.	2018	A.C. Camargo Cancer Center and Barretos Cancer Hospital, SP	NA	40	PCR	57%
De Carvalho et al.	2019	HNS Department at the Barretos Cancer Hospital, SP	2006-2012	25	IHQ	20%
De Cicco et al.	2019	A.C. Camargo Cancer Center, SP	1984-2014	215	IHC, PCR	59%
Caldeira et al.	2020	HNS Department of Clinics Hospital (UFMG) and Hospital da Baleia, MG	2005-2015	23	IHC	13%
De Carvalho et al.	2020	HNS Department at the Barretos Cancer Hospital, SP	2009-2017	51	IHC, PCR	IHC: 35% (n=51) | PCR: 50% (n=12)
Girardi et al.	2020	HNS Department of Clinics Hospital (UFRGS), RS	2017-2019	91	IHC	21%
Buexm et al.	2020	Brazilian National Cancer Institute, RJ	1999-2010	346	IHC, PCR	IHC - 9% | PCR - 12% IHC+PCR = 6%
Matos et al.	2020	Cancer Institute of São Paulo (ICESP), SP	2009-2018	60	IHC	13%
Cury et al.	2021	A.C. Camargo Cancer Center, SP	1998-2017	10	PCR	40%
Da Silva Santos et al.	2021	Araçatuba Dental School, São Paulo State University (UNESP), SP	NA	9	PCR	0%
De C. Ferreira et al.	2021	HNS Department at the Barretos Cancer Hospital, SP	2014-2019	252	IHC	32%
De Oliveira Filho et al.	2021	Haroldo Juaçaba Hospital/Cancer Institute of Ceará, CE	2006-2018	50	IHC	42%
Santos-Carvalho et al.	2021	HNS Department at the Barretos Cancer Hospital, SP	2008-2018	792	IHQ	21%
Schiavetto et al.	2021	HNS Department at the Barretos Cancer Hospital, SP	2007-2018	50	IHQ, PCR	IHQ: 32% | PCR: 42% IHC+PCR= 26%
Silveira et al.	2021	Pathology Center of School of Medicine (USP), SP	NA	126	IHC, ISH	IHC: 23% | ISH: 25% IHC+ISH=23%
Gama-Cuellar et al.	2022	HNS Department at the A.C. Camargo Cancer Center, SP	2015-2020	33	IHC	22%
Pires et al.	2022	HNS Department at the Barretos Cancer Hospital, SP	2013-2017	254	IHC	32%
Sichero et al.	2022	Cancer Institute of São Paulo (ICESP), SP	2015-2019	66	IHQ, PCR	IHQ: 19% | PCR: 11% IHC+PCR= 3%
Present study	2023	Oral Pathology Center, School of Dentistry (UFMG), MG	1997-2021	146	IHC, ISH	IHC: 13% | ISH: 13% IHC+ISH=13%

IHC: Immunohistochemistry for p16; PCR: Polymerase chain reaction; ISH: In situ hybridization; IF: Immunofluorescence for p16; NA: not available; HNS: Head and neck surgery; USP: Universidade de São Paulo; FAMERP: Faculdade de Medicina de São José do Rio Preto; UNICAMP: Universidade Estadual de Campinas; UNESP: Universidade Estadual Paulista; UFMG: Universidade Federal de Minas Gerais; UFRGS: Universidade Federal do Rio Grande do Sul; SP: São Paulo; RJ: Rio de Janeiro; GO: Goiás; MG: Minas Gerais; RS: Rio Grande do Sul; ES: Espírito Santo; DF: Distrito Federal; CE: Ceará; MT: Mato Gosso.
